# Associations between Meat and Vegetable Intake, Cooking Methods, and Asthenozoospermia: A Hospital-Based Case–Control Study in China

**DOI:** 10.3390/nu14091956

**Published:** 2022-05-07

**Authors:** Ya-Shu Liu, Yi-Xiao Zhang, Xiao-Bin Wang, Qi-Jun Wu, Fang-Hua Liu, Bo-Chen Pan, Yu-Hong Zhao

**Affiliations:** 1 Department of Clinical Epidemiology, Shengjing Hospital of China Medical University, Shenyang 110004, China; yashu_liu@126.com (Y.-S.L.); wuqj@sj-hospital.org (Q.-J.W.); liufanghua5865@163.com (F.-H.L.); 2 Clinical Research Center, Shengjing Hospital of China Medical University, Shenyang 110004, China; 3 Department of Urology, Shengjing Hospital of China Medical University, Shenyang 110004, China; zhangyx@sj-hospital.org; 4 Center for Reproductive Medicine, Shengjing Hospital of China Medical University, Shenyang 110004, China; wangxb@sj-hospital.org

**Keywords:** asthenozoospermia, meat, vegetable, cooking methods, case–control study

## Abstract

**Background:** The role of meat and vegetable intake in the development of asthenozoospermia has been controversial, and the role of cooking methods for meat and vegetables in the association has yet to be determined. The present study aimed to illuminate the relationship between the consumption and cooking methods of meat and vegetables and the risk of asthenozoospermia. **Methods:** In this hospital-based case–control study, we enrolled 552 patients with asthenozoospermia and 585 healthy controls. Dietary information was assessed using a validated self-administered food frequency questionnaire. Asthenozoospermia was diagnosed according to the fifth edition of the WHO laboratory manual for the examination and processing of human semen. **Results:** Participants in the highest tertile of total meat and unprocessed meat intake had a 44% and 39% lower risk of asthenozoospermia than those in the lowest tertile (OR = 0.56, 95% CI: 0.37, 0.87 and OR = 0.61, 95% CI: 0.40, 0.93), respectively. Participants with the highest processed meat consumption showed higher risk (OR = 1.44, 95% CI: 1.01, 2.06). Raw vegetable consumption was negatively associated with the risk of asthenozoospermia (OR = 0.67, 95% CI: 0.45, 0.98). The stir-frying cooking method for meat was associated with increased risk of asthenozoospermia (OR = 1.58, 95% CI: 1.02, 2.46). **Conclusions:** Intake of total meat, unprocessed meat, and raw vegetable may reduce asthenozoospermia risk, while higher consumption of processed meat may increase the risk. Cooking methods may play a role in these associations. These findings need to be confirmed in large and prospective cohort studies.

## 1. Introduction

Asthenozoospermia is characterized by reduced motility or a total lack of sperm motility in a fresh ejaculate [1]. Motility is required for sperm to migrate from the vagina to the fallopian tubes and penetrate the zona pellucida of the egg [2,3]. More than 40% of infertile men exhibit asthenozoospermia [4], and 24% of infertile cases exhibit isolated asthenozoospermia [5]. A previous study estimated that the sperm count in U.S. males is decreasing by 1.5% annually [6]. In addition, the sperm concentration has decreased by over 50% in Western countries from 1973 to 2011 [7]. Therefore, it is of great significance to investigate the factors that underlie this disease to improve male reproductive health. Previous studies have established several well-known risk factors for asthenozoospermia including sperm dysfunction (such as low sperm motility and lower semen quality scores), prolonged period of sexual abstinence, varicocele, infections (such as viral infections and even the recent COVID-19 pandemic), and genetic factors [8,9,10,11,12,13,14,15]. In addition, lifestyle patterns may also play an important role in the development of asthenozoospermia [16]. Of note, compared with these factors, dietary habits may be more modifiable by preventive interventions.

Previous studies have suggested that fruits, nuts and whole cereals, fish, seafood, poultry, and low-fat dairy product intake are positively associated with sperm quality. However, diets high in lipophilic foods, soy isoflavones, and sweets have been shown decreasing the semen quality [17,18]. Furthermore, animal studies showed that high-fat diet, vitamin D deficiency, and hypercholesterolemic diet were associated with poor sperm quality, and simultaneous zinc (Zn) supplementation with iron and olive oil were found to be the protective factors for sperm quality [19,20,21,22]. However, for asthenozoospermia, although many studies have investigated the associations between diverse food items and its risk [23,24,25,26], the conclusions derived from these studies remain controversial. For example, whereas two observational studies suggested that poultry intake was associated with a decreased risk of asthenozoospermia and increased fertilization rates, and intake of processed meat was inversely associated with the reproductive outcomes [23,26], several cross-sectional studies failed to demonstrate any associations between the same types of exposure and the progressive motility of sperm [24,25]. We speculate that, in addition to differences in study design and sample size, the inconsistency and heterogeneity that are evident in the existing literature may also be attributed to the potential modifying effect of cooking methods, which were not considered in most of these previous studies [23,24,25,26]. The modifying effect created by different cooking methods has important clinical significance because of the formation of unfavorable heterocyclic amines (HCAs) [27] and changes in the phytochemical content [28] during cooking. However, to our knowledge, few studies have considered the association between meat and vegetable intake and the risk of asthenozoospermia in relation to their cooking methods.

Herein, we conducted a hospital-based case–control study to specifically determine whether there were any associations between intake of meat and vegetables, cooking methods, and the risk of asthenozoospermia.

## 2. Materials and Methods

### 2.1. Participants

The participants of this case–control study were recruited from the infertility clinic at Shengjing Hospital of China Medical University in Liaoning Province, China, between June 2020 and December 2020. In total, 643 asthenozoospermia patients and 662 healthy controls were recruited. All participants received health examinations and were asked to complete a structured and self-administered health status questionnaire. The questionnaire covered questions relating to marital status, employment status, educational level, physical activity, sleep habits, and dietary habits. The study was approved by the ethics committee of Shengjing Hospital of China Medical University (2017PS190K) and was performed in accordance with the ethical standards laid down in the 1964 Declaration of Helsinki and its later amendments. All participants provided written informed consent prior to study inclusion.

**Figure 1:**
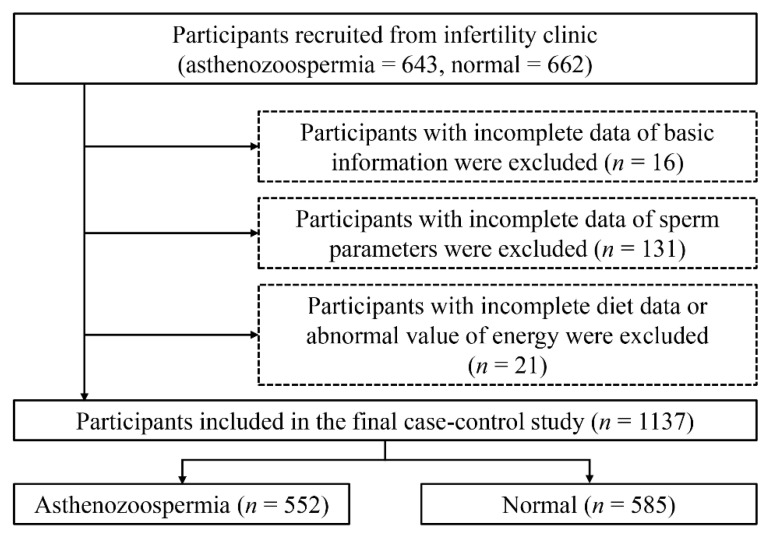
Flow-chart of the selection of participants.

As shown in Figure 1, we excluded participants who failed to provide detailed information on sperm parameters (*n* = 131) or other basic information (physical activity, weight, high, smoking status, drinking status, income, and educational level) (*n* = 16). We also excluded those who failed to record their food consumption and cooking habits or provide abnormal values for total energy intake (>6000 kcal or <800 kcal) (*n* = 21) [29].

Finally, 552 cases and 585 controls were included in this case–control study.

### 2.2. Assessment of Dietary Data

Dietary intake was assessed using an original food frequency questionnaire (FFQ) that included 110 food items and 21 items related to dietary habit (including cooking methods for meat and total vegetable). We asked participants to recall the average frequency of dietary intake in the past year. The FFQ included seven frequency categories ranging from ‘almost never’ to ‘twice or more per day’ for foods. The reproducibility and validity of the questionnaire was assessed in a random sample of 150 participants living in the Northeast China Region by using data from repeated measurements of the FFQ approximately three months apart and weight diet records (WDRs) over a four-day period. The spearman correlation coefficients and intraclass correlation coefficients for reproducibility were above 0.5 for most food groups, and the correlation coefficients were 0.3–0.7 for most food groups between the FFQ and WDRs [30].

The mean daily intake of food and nutrients (such as meat, total vegetable, and total energy) were calculated by using an ad hoc computer program developed to specifically analyze the questionnaire. The intake of food items was calculated from portion size (g/time) and the frequency of each food item consumed per day. We calculated the amount of each food in the food group on the basis of data from the twice 4-day WDRs and defined the medium value of each food as the portion size of each food. Furthermore, valid and reliable Chinese food composition tables [31] were used as a nutrient database to determine the nutrient content of each food item. Processed meat was defined as processed meatballs, sausage, ham, bacon, and bologna. Nutrient intake was calculated by first multiplying the consumption for each food item (in grams) by its nutrient content (per gram) and then adding the nutrient contributions across all food items. For cooking methods, participants were asked to select their most frequent method of cooking meat (steaming, stewing, broiling, deep-frying, and stir-frying) and total vegetable (raw, stewing, broiling, deep-frying, and stir-frying) following seven frequency categories: almost never, 2−3 times/month, 1 time/week, 2–3 times/week, 4–6 times/week, 1 time/day, and ≥2 times/day.

### 2.3. Assessment and Definition of Asthenozoospermia

The rationale and details of semen analysis and definition of asthenozoospermia have been reported in previous studies [32,33,34], and the checklist for acceptability of studies based on human semen analysis is shown in Suppl. A [35].Moreover, guidelines provided by the World Health Organization (WHO) were used to classify the sperm motility. Asthenozoospermia was defined by the fifth edition of the WHO laboratory manual for the examination and processing of human semen [36] (details shown in Suppl. B). Controls were normozoospermic men (≥15 × 10^6^ sperm/mL, ≥40% total motility, ≥32% progressive motility, and ≥4% normal forms) attending the same infertility clinics as the cases.

### 2.4. The Assessment and Definition of Other Variables

Sociodemographic variables, including age, education, income, mental condition, and physical activity, were assessed by a questionnaire survey. Information on the smoking status (never, former, or current smoker) and drinking status (never, former, or current drinking) of the participants was obtained from the questionnaire survey. Educational level was divided into three categories: junior secondary or below, senior high school/technical secondary school, and junior college/university or above. Annual income level was divided into three categories: less than RMB 50,000, RMB 50,000–100,000, and more than RMB 100,000. Metabolic equivalent (MET) hours per week were calculated using corresponding MET coefficients that have been described in detail previously [37]. Physical examination data were also collected, including height and weight. Body mass index (BMI) was calculated as weight in kilograms divided by the square of height in meters (kg/m^2^).

### 2.5. Statistical Analysis

The normality of all continuous variables was evaluated through the Kolmogorov–Smirnov statistic test. The baseline characteristics of the study participants are presented herein according to asthenozoospermia status. Continuous and categorical variables are presented as mean ± standard deviation and percentages and were tested with the independent sample Student’s *t*-test and the chi-squared test, respectively. The final categories for the frequency of different cooking methods for meat and total vegetable according to the frequency distribution of the responses were as follows: less than 2–3 times per month, 1–3 times per week, and more than 4 times per week. Tertiles were categorized across the consumption of unprocessed meat, processed meat, and total vegetables and used for further analyses. Associations between dietary factors (intake of meat, total vegetables, and different cooking methods) and asthenozoospermia were evaluated by unconditional logistic regression analysis. Odds ratios (ORs) and 95% confidence intervals (CIs) were also calculated. The linear trend cross increasing tertiles was tested using the median value of each quartile as a continuous variable based on logistic regression. 

Model 1 was adjusted for age (years) and BMI (kg/m^2^). Model 2 was adjusted for age, BMI, smoking status (yes/no), drinking status (yes/no), household income (RMB; thousand yuan), abstinence time (days) [36,38,39], educational level (junior secondary or below, senior high school/technical secondary school, and junior college/university or above), total energy intake (kcal/day), and physical activity (MET/hours/week). For total meat, model 3 was further adjusted for different cooking methods (times/day) and total vegetable intake (g/day). For unprocessed meat, model 3 was further adjusted for different cooking methods (times/day), processed meat intake (g/day), and total vegetable intake (g/day). For processed meat, model 3 was further adjusted for different cooking methods (times/day), unprocessed meat intake (g/day), and total vegetable intake (g/day). For total vegetable, model 3 was further adjusted for different cooking methods (times/day) and total meat intake (g/day). For meat cooking methods analysis, model 3 was further adjusted for vegetable cooking methods (times/day), total meat intake, and total vegetable intake (g/day). For the vegetable cooking method, model 3 was further adjusted for meat cooking methods (times/day), total meat intake, and total vegetable intake (g/day).

We also carried out subgroup analyses according to age (<32 years or ≥32 years), BMI level (<25 kg/m^2^ or ≥25 kg/m^2^), and smoking status (never-smokers or ever-smokers). We also tested interaction effects between dietary factors (intake of unprocessed meat, processed meat, total vegetables, and different cooking methods for meat and total vegetables), and age/BMI/smoking on asthenozoospermia. The multiplicative term of dietary factors and age, dietary factors and BMI, or dietary factors and smoking status, when adjusted for all confounding factors, were also calculated to test the significance of interaction effects. All tests were two-sided, with *p* < 0.05 considered to be statistically significant. Statistical analyses were performed using SAS version 9.3 (SAS Institute, Cary, NC, USA) (programs of main analysis shown in Suppl. C).

## 3. Results

### 3.1. Participant Characteristics

The specific characteristics of cases and controls are presented in Table 1. Cases of asthenozoospermia tended to be older, non-drinkers, and had a longer abstinence time. Furthermore, such subjects had a lower sperm concentration, total sperm count, progressive motility, and total motility. They also exhibited less normal sperm morphology and consumed less total and unprocessed meat (more details shown in Suppl. D).

### 3.2. Meat Consumption, Cooking Method, and Asthenozoospermia Risk

Participants in the highest tertile of total meat and unprocessed meat intakes had a 44% and 39% lower risk of asthenozoospermia than those in the lowest tertile (OR = 0.56, 95% CI: 0.37, 0.87; *p*-trend < 0.05 and OR = 0.61, 95% CI: 0.40, 0.93; *p*-trend < 0.05), respectively. However, participants with the highest consumption of processed meat had a higher risk of asthenozoospermia (OR = 1.44, 95% CI: 1.01, 2.06) (Table 2). Significant associations between these factors were also observed in the subgroup analyses when stratified by age, BMI, and smoking status (Figure S1, Table S1). Moreover, we found stir-fried meat was associated with an increased risk of asthenozoospermia in model 2 (OR = 1.58, 95% CI: 1.02, 2.46) (Table 3). However, other cooking methods for meat were not significantly associated with asthenozoospermia (Table 3 and Table S2). The same results were observed in the interaction analysis of meat intake and cooking methods with asthenozoospermia risk (Table S3).

### 3.3. Vegetable Consumption, Cooking Method, and the Risk of Asthenozoospermia 

Although total vegetable intake was not associated with the risk of asthenozoospermia in all participants (Table 2), we observed an inverse association in participants whose BMI was ≥25 kg/m^2^ (Supplementary Figure S1, Table S1). In addition, compared with participants in the lowest tertile, the highest tertile of raw vegetable consumption was negatively associated with the risk of asthenozoospermia (OR = 0.67, 95% CI: 0.45, 0.98, *p*-trend < 0.05) (Table 4). We did not identify any significant associations between total vegetable intake, cooking methods and the risk of asthenozoospermia in our interaction analyses (Table S4).

## 4. Discussions

To the best of our knowledge, this is the first study to investigate the roles of cooking methods in the association between meat and vegetable intake and asthenozoospermia. Our study showed that intake of total meat, unprocessed meat, and raw vegetables was associated with a reduced risk of asthenozoospermia, while the intake of processed meat and stir-fried meat was associated with an increased risk of this disease.

Our findings that total and unprocessed meat intake were associated with a reduced risk of asthenozoospermia were in line with some previous studies. For example, two case–control studies highlighted the protective effects of red meat and poultry intake on asthenozoospermia and sperm quality [23,40]. A cohort study involving 141 men also demonstrated a positive association between poultry intake and fertilization rates [26]. However, several studies failed to verify the significance of these associations [24,25,41,42,43,44]. We speculate that this might be attributed to limited sample size of these studies. In addition, difference in age and race of the participants may also have contributed to the inconsistencies. For example, compared with our study (mean age = 33.29 years), participants of studies in the United States (mean age = 19.8 years) and Spain (median age = 20.5 years) were relatively younger [25,41]. On the other hand, results on the relationship between processed meat and sperm quality has been relatively consistent. For instance, a previous case–control study showed that processed meat intake was significantly associated with high risk of asthenozoospermia after adjusting for potential confounders [23]. Moreover, other previous studies demonstrated that participants with low sperm quality tended to consume more processed meat [42,43,45]. In this sense, our finding on the positive association between consumption of processed meat and increased risk of asthenozoospermia was supporting these published studies [23,42,43,45], further illustrating the adverse effect of processed meat on sperm quality.

The cooking methods for meat in relation to asthenozoospermia have not been reported before. Our current study has identified a positive association between the meat cooked by stir-frying and the risk of asthenozoospermia. This finding is important in a couple of ways. First, although our study has demonstrated that higher intake of total meat or the unprocessed meat is beneficial to sperm motility, improper cooking such as stir-frying could change the protective role of the meat and reduce or eliminate such beneficial effect. It reminds us that proper cooking method for meat is needed to maintain its role for a healthy diet. Second, our study has shown that the most frequent method of cooking for meat was stir-frying (Table 3), indicating that our patients or population should be educated about the healthy way of cooking the meat. In this regard, it might be interesting to note that our study also found (albeit not statistically significant) that steaming tended to represent a potentially useful means of protecting nutrients of the meat and a relatively healthy cooking method [46]. However, further study is needed to confirm this finding. 

Similarly, inconsistency in study findings on the relationship between vegetable intake and sperm quality also exists. Our study failed to identify a significant association between total vegetable intake and the risk of asthenozoospermia, supporting the results of some previous studies [41,47,48]. However, a case–control study in Iran, including 72 asthenozoospermic cases and 169 normozoospermic controls, found that a high intake of vegetables was significantly associated with a reduced risk of asthenozoospermia [23]. Moreover, a cross-sectional study conducted in Rotterdam, involving 161 men, showed that the intake of vegetables was positively correlated with sperm quality [44]. We speculate that these inconsistencies may also be partly due to different population characteristics (e.g., age, income level, and physical activity) and the sample size. In this regard, it might be important to note that participants of one previous study were patients undergoing in vitro fertilization treatment, which may constitute a different population from subjects in our study [41]. Finally, regarding the cooking method for vegetables, similar to our results, one case–control study conducted in Spain also found that raw vegetable intake was associated with better sperm quality [43]. Therefore, it might be beneficial to encourage patients to increase the raw vegetable component of their diet.

Meat is a natural source of Zn elements [44]. Zn acts as a membrane-stabilizing source by inhibiting membrane-bound oxidative enzymes such as NAD(p) oxidase [49], thus increasing sperm motility [50]. However, consumption of processed meat is known to have a detrimental effect on asthenozoospermia; this may stem from the presence of preservatives and more residues of active substances (such as exogenous estrogens) [51,52,53,54]. Previous experimental research found that preservatives in the food reduced the synthesis of androgens, which led to decreased sperm production and function in the treated male rats. This effect could be mediated by a decreased synthesis of lutenizing hormone and follicle-stimulating hormone from the pituitary gland caused by the disruption of the hypothalamo–pituitary–gonadal axis [55]. Moreover, cooking processes that involve water inevitably result in the leaching of nutrients (e.g., Zn) from meat into the cooking medium [56,57]. In addition, unfavorable heterocyclic amine (HCAs) may be formed during the cooking processes [58], and further formation of reactive species during HCA metabolism may result in oxidative stress, causing cellular damage and the loss of biological function [59]. Indeed, one animal experiment showed that HCAs had strong testis toxicity in F344 male rats [60]. On the other hand, the mechanisms that have been proposed to explain the protective role of raw vegetables is also strongly related to the high vitamin content in the vegetables. At high temperature and over a long period of time, just as in the process of cooking, there is large amount of degradation of heat-sensitive micronutrients, such as vitamin C, folates, and thiamine [61]. Therefore, consumption of raw vegetables may avoid the loss of vitamins and may have a more significant protective effect on asthenozoospermia.

There are several limitations in our study that should be noted. Recall bias and selection bias existed due to the nature of the self-reporting questionnaire form used in this hospital-based case–control study. In addition, it is impossible to infer causality by a case-control study design, and reverse causality cannot be avoided. Therefore, large and prospective cohort studies and animal experiments are needed to confirm our findings. Moreover, only a few participants reported some cooking methods as the most frequently used in subgroup analyses (e.g., broiling meat in the cohort of smokers). Therefore, the statistical power of our study may not have been sufficient to detect a significant association. The results derived from our subgroup analyses should be interpreted with caution due to the limited sample size in some categories. Furthermore, although we adjusted for a considerable number of potential confounding factors, residual or unmeasured confounding may still exist (such as psychological stress, physical stress, and type of jobs). In addition, due to geographical and its related dietary reasons, our findings should be interpreted with caution. Finally, the use of antibiotics could be a confounding factor and potentially responsible for the inconsistencies between the studies on meat products. Previous studies showed that antibiotic residues in food had adverse reproductive effects [18,62]. However, due to the limitation of data, we did not estimate the effect of antibiotic residues.

Nevertheless, this is the first case–control study to explore the roles of intake content and cooking methods for meat and vegetables in relation to the risk of asthenozoospermia, and significant results have been found. The strengths of our study include the use of a validated FFQ to collect detailed information about habitual food intake. Moreover, compared with previous studies, our large sample size and high participation rates were advantageous in reducing the random errors that are often associated with studies with low participant numbers. Furthermore, we adjusted a considerable number of potential confounders, such as sociodemographic characteristics, lifestyle factors, and other dietary information.

## 5. Conclusions

Our study showed that the intake of total meat, unprocessed meat, and raw vegetables was associated with reduced risk of asthenozoospermia, whereas consumption of processed meat was associated with increased risk of asthenozoospermia. In addition, certain cooking style for meat such as stir-frying was also associated with increased risk of asthenozoospermia. These findings suggest that increasing the intake of unprocessed meat and raw vegetables, decreasing the intake of processed meat, and using the right cooking method for meat may be beneficial to the sperm motility. Further large and prospective cohort studies are needed to confirm these findings.

## Figures and Tables

**Table 1:** Characteristics of participants according to asthenozoospermia status.

Characteristics	Normal	Asthenozoospermia	*p*-Value
No. of participants	585	552	
Age (years)	32.12 ± 4.50	33.29 ± 5.26	**<0.05**
Body mass index (kg/m^2^)	26.25 ± 4.55	26.41 ± 4.41	0.529
Physical activity (MET/hours/week)	166.50 ± 103.04	166.77 ± 103.08	0.965
Abstinence time (days)	4.28 ± 1.39	4.48 ± 1.48	**<0.05**
**Semen parameters**			
Ejaculate volume (mL)	3.45 ± 1.26	3.62 ± 1.48	**<0.05**
Sperm concentration (10^6^/mL)	71.09 ± 39.76	58.84 ± 36.09	**<0.05**
Total sperm count (10^6^/mL)	232.83 ± 133.59	199.68 ± 126.12	**<0.05**
Progressive motility (%)	44.62 ± 9.33	22.05 ± 8.72	**<0.05**
Total motility (%)	55.00 ± 11.35	27.97 ± 10.92	**<0.05**
Normal sperm morphology (%)	6.66 ± 2.72	5.71 ± 2.54	**<0.05**
**Smoking status (%)**			
No	275 (47.01)	287 (51.99)	0.093
Yes	310 (52.99)	265 (48.01)	
**Drinking status (%)**			
No	333 (56.92)	352 (63.77)	**<0.05**
Yes	252 (43.08)	200 (36.23)	
**Educational level (%)**			
Junior secondary or below	143 (24.44)	121 (21.92)	0.603
Senior high school/technical secondary school	843 (14.19)	79 (14.31)	
Junior college/university or above	359 (61.37)	352 (63.77)	
**Annual family income (RMB thousand yuan) (%)**			
<50	94 (16.07)	99 (17.93)	0.698
50 to <100	229 (39.15)	210 (38.04)	
≥100	262 (44.79)	243 (44.02)	
**Diet**			
Energy (kcal/d)	1781.26 ± 596.48	1844.26 ± 633.14	0.084
Total meat (g/d)	106.79 ± 48.77	100.50 ± 46.23	**<0.05**
Unprocessed meat (g/d)	100.33 ± 47.48	93.24 ± 44.58	**<0.05**
Processed meat (g/d)	6.46 ± 7.36	7.26 ± 8.75	0.093
Total vegetable (g/d)	196.58 ± 135.34	210.86 ± 151.94	0.094
**Cooking methods**			
Deep-frying for meat (times/month)	3.29 ± 4.27	2.71 ± 3.67	**<0.05**
Stewing for meat (times/month)	10.27 ± 7.88	9.66 ± 7.45	0.227
Broiling for meat (times/month)	3.25 ± 3.02	3.24 ± 3.82	0.782
Stir-frying for meat (times/month)	22.01 ± 12.17	20.72 ± 11.98	**<0.05**
Steaming for meat (times/month)	1.69 ± 3.05	1.84 ± 3.31	0.403
Deep-frying for total vegetable (times/month)	1.34 ± 4.17	1.26 ± 3.64	0.740
Stewing for total vegetable (times/month)	11.94 ± 11.47	12.53 ± 11.26	0.302
Broiling for total vegetable (times/month)	2.11 ± 3.94	1.74 ± 3.21	0.113
Stir-frying for total vegetable (times/month)	22.97 ± 14.55	24.86 ± 15.64	0.062
Raw vegetables (no cooked) (times/month)	6.08 ± 7.58	7.75 ± 9.92	**<0.05**

Data were presented in mean ± standard deviation or count (percentage), and *p*-value was the results from analysis of independent sample Student’s t-tests or chi-square tests where appropriate.

**Table 2:** Associations between meat and total vegetable intake and the asthenozoospermia risk.

	Consumption of Meat and Vegetables			*p*-Trend ^*^
	Level 1	Level 2	Level 3	
**Total Meat (g/d)**	≤89.43	89.43–116.44	>116.44	
Case/control	222/193	160/189	170/203	
Model 1 ^a^	1.00 (reference)	**0.72 (0.54, 0.97) ^†^**	**0.75 (0.56, 0.99)**	**0.010**
Model 2 ^b^	1.00 (reference)	**0.69 (0.51, 0.92)**	**0.63 (0.46, 0.86)**	**0.002**
Model 3 ^c^	1.00 (reference)	**0.63 (0.43, 0.91)**	**0.56 (0.37, 0.87)**	**0.008**
**Unprocessed meat (g/d)**	≤85.68	85.68–108.56	>108.56	
Case/control	190/173	117/116	245/296	
Model 1 ^a^	1.00 (reference)	**0.91 (0.65, 1.27)**	**0.75 (0.57, 0.98)**	**0.039**
Model 2 ^b^	1.00 (reference)	**0.89 (0.63, 1.24)**	**0.66 (0.49, 0.88)**	**0.006**
Model 3 ^c^	1.00 (reference)	**0.83 (0.55, 1.24)**	**0.61 (0.40, 0.93)**	**0.021**
**Processed meat (g/d)**	≤2.87	2.87–5.74	≥5.74	
Case/control	96/116	196/207	260/262	
Model 1 ^a^	1.00 (reference)	1.31 (0.93, 1.85)	**1.45 (1.04, 2.04)**	**0.048**
Model 2 ^b^	1.00 (reference)	1.35 (0.96, 1.92)	1.41 (0.99, 2.01)	0.111
Model 3 ^c^	1.00 (reference)	1.41 (0.99, 2.01)	**1.44 (1.01, 2.06)**	0.112
**Total vegetable (g/d)**	≤121.25	121.25–207.07	>207.07	
Case/control	174/194	179/195	199/196	
Model 1 ^a^	1.00 (reference)	1.03 (0.77, 1.37)	1.13 (0.85, 1.50)	0.399
Model 2 ^b^	1.00 (reference)	0.95 (0.70, 1.28)	0.91 (0.64, 1.27)	0.576
Model 3 ^c^	1.00 (reference)	0.94 (0.69, 1.27)	0.83 (0.58, 1.18)	0.299

^*^ Analysis of multiple logistic regression. ^†^ Odds ratio (95% confidence interval) (all such value). ^a^ Adjusted for age and BMI. ^b^ Adjusted for age, BMI, smoking status, drinking status, total energy intake, household income, abstinence time, educational level, and physical activity. ^c^ Further adjusted for cooking methods, total meat intake, unprocessed meat intake, processed meat intake, and total vegetable intake (based on model 2) (mutually adjusted for one another).

**Table 3:** Associations between different cooking methods for meat and asthenozoospermia.

	Frequency of Different Cooking Methods for Meat			*p*-Trend ^*^
	2–3 Times/Month	2~3 Times/Week	4 Times/Week	
**Deep-frying**				
Case/control	428/437	113/133	11/15	
Model 1 ^a^	1.00 (reference)	1.17 (0.52, 2.71) ^†^	1.29 (0.59, 2.94)	0.409
Model 2 ^b^	1.00 (reference)	1.21 (0.53, 2.86)	1.41 (0.62, 3.28)	0.286
Model 3 ^c^	1.00 (reference)	0.98 (0.42, 2.34)	1.10 (0.48, 2.61)	0.655
**Stewing**				
Case/control	132/126	328/349	92/110	
Model 1 ^a^	1.00 (reference)	1.14 (0.83, 1.56)	1.26 (0.87, 1.83)	0.268
Model 2 ^b^	1.00 (reference)	1.12 (0.88, 1.70)	1.38 (0.93, 2.04)	0.125
Model 3 ^c^	1.00 (reference)	0.96 (0.67, 1.38)	0.94 (0.59, 1.49)	0.802
**Broiling**				
Case/control	425/452	122/128	5/5	
Model 1 ^a^	1.00 (reference)	1.01 (0.27, 3.72)	0.97 (0.27, 3.52)	0.835
Model 2 ^b^	1.00 (reference)	1.06 (0.28, 3.94)	0.98 (0.27, 3.62)	0.765
Model 3 ^c^	1.00 (reference)	0.88 (0.23, 3.35)	0.79 (0.21, 3.00)	0.515
**Stir-frying**				
Case/control	56/41	135/148	361/396	
Model 1 ^a^	1.00 (reference)	1.00 (0.76, 1.32)	1.47 (0.96, 2.28)	0.340
Model 2 ^b^	1.00 (reference)	1.07 (0.80, 1.42)	**1.58 (1.02, 2.46)**	0.161
Model 3 ^c^	1.00 (reference)	0.84 (0.61, 1.16)	1.09 (0.67, 1.81)	0.522
**Steaming**				
Case/control	471/503	76/78	5/4	
Model 1 ^a^	1.00 (reference)	0.89 (0.21, 3.53)	0.84 (0.21, 3.23)	0.700
Model 2 ^b^	1.00 (reference)	0.84 (0.20, 3.37)	0.85 (0.21, 3.31)	0.859
Model 3 ^c^	1.00 (reference)	0.78 (0.18, 3.23)	0.73 (0.17, 3.00)	0.609

^*^ Analysis of multiple logistic regression. ^†^ Odds ratio (95% confidence interval) (all such value). ^a^ Adjusted for age and BMI. ^b^ Adjusted for age, BMI, smoking status, drinking status, total energy intake, household income, abstinence time, educational level, and physical activity. ^c^ Further adjusted for total meat intake, total vegetable intake, and different cooking methods for total vegetable (based on model 2).

**Table 4:** Association between different cooking methods for total vegetables and asthenozoospermia.

	Frequency of Different Cooking Methods for Total Vegetables			*p*-Trend ^*^
	2–3 Times/Month	2~3 Times/Week	4 Times/Week	
**Deep-frying for total vegetable**				
Case/control	508/531	34/45	10/9	
Model 1 ^a^	1.00 (reference)	0.66 (0.24, 1.82) ^†^	0.83 (0.32, 2.08)	0.923
Model 2 ^b^	1.00 (reference)	0.82 (0.29, 2.30)	1.07 (0.40, 2.79)	0.669
Model 3 ^c^	1.00 (reference)	0.98 (0.34, 2.84)	1.28 (0.48, 3.44)	0.441
**Stewing for total vegetable**				
Case/control	119/137	254/289	179/159	
Model 1 ^a^	1.00 (reference)	0.78 (0.59, 1.03)	0.79 (0.57, 1.09)	0.065
Model 2 ^b^	1.00 (reference)	0.79 (0.60, 1.05)	0.85 (0.60, 1.19)	0.140
Model 3 ^c^	1.00 (reference)	0.76 (0.58, 1.01)	0.82 (0.58, 1.15)	0.086
**Broiling for total vegetable**				
Case/control	486/496	58/76	8/13	
Model 1 ^a^	1.00 (reference)	1.51 (0.45, 3.09)	1.44 (0.60, 3.67)	0.255
Model 2 ^b^	1.00 (reference)	1.43 (0.54, 4.10)	1.85 (0.73, 5.13)	0.116
Model 3 ^c^	1.00 (reference)	1.55 (0.57, 4.59)	1.97 (0.75, 5.67)	0.107
**Stir-frying for total vegetable**				
Case/control	30/38	131/148	391/399	
Model 1 ^a^	1.00 (reference)	0.92 (0.70, 1.21)	0.84 (0.51, 1.39)	0.416
Model 2 ^b^	1.00 (reference)	0.98 (0.74, 1.30)	0.93 (0.55, 1.54)	0.790
Model 3 ^c^	1.00 (reference)	0.97 (0.73, 1.28)	0.91 (0.54, 1.53)	0.724
**Raw vegetable**				
Case/control	265/315	198/201	89/69	
Model 1 ^a^	1.00 (reference)	0.76 (0.52, 1.10)	**0.66 (0.46, 0.95)**	**0.025**
Model 2 ^b^	1.00 (reference)	0.78 (0.53, 1.14)	0.69 (0.48, 1.00)	0.054
Model 3 ^c^	1.00 (reference)	0.75 (0.51, 1.11)	**0.67 (0.45, 0.98)**	**0.041**

^*^ Analysis of multiple logistic regression. ^†^ Odds ratio (95% confidence interval) (all such value). ^a^ Adjusted for age and BMI. ^b^ Adjusted for age, BMI, smoking status, drinking status, total energy intake, household income, abstinence time, educational level, and physical activity. ^c^ Further adjusted for total meat intake, different cooking methods for meat, and total vegetable intake (based on model 2).

## Data Availability

The original contributions presented in the study are included in the article/Supplementary Material; further inquiries can be directed to the corresponding authors.

## Supplementary Materials

The following supporting information can be downloaded at https://www.mdpi.com/article/10.3390/nu14091956/s1. Suppl. A–D, Supplementary Figure S1, and Supplementary Tables 1–5. Figure S1. Subgroup analysis of association between food intake and asthenozoospermia by age, BMI, and smoking status. Analysis of multiple logistic regression, adjusted for age, BMI, smoking status, drinking status, household income, abstinence time, educational level, physical activity, total energy intake, different cooking methods, total meat intake, unprocessed meat intake, processed meat intake, and total vegetable intake (mutually adjusted for one another). Table S1. Subgroup analysis of association between food intake and asthenozoospermia by age, BMI, and smoking status. Table S2. Subgroup analysis of association between different cooking methods and asthenozoospermia by age, BMI, and smoking status. Table S3. Odds ratio (95% CI) for risk of asthenozoospermia according to combined effect of meat intake and cooking methods. Table S4. Association between different cooking methods for vegetables and asthenozoospermia. Table S5. Odds ratio (95% CI) for risk of asthenozoospermia according to combined effect of vegetables intake and cooking methods. Suppl. A. The checklist for acceptability of studies based on human semen analysis. Suppl. B The examination and processing of human semen. Suppl. C. SAS programs of main analysis. Suppl. D. Different types meat intake and association between total fish intake and asthenozoospermia.
