# Supplementary material for: Associations between Meat and Vegetable Intake, Cooking Methods, and Asthenozoospermia: A Hospital-Based Case–Control Study in China
Source: Nutrients. 2022 May 7;14(9):1956. doi: 10.3390/nu14091956 (PMC9104795; doi:10.3390/nu14091956)
Supplement: Supplementary file 1 [file nutrients-14-01956-s001.zip › nutrients-1661098-supplementary/Supplementary/supplA-D.pdf]

Suppl. A

Patients

☑ For clinical studies: The patient population (e.g. patients, volunteers, students) has been declared in the manuscript, together with the recruitment method and inclusion and exclusion criteria. If the study concerns couples being investigated for infertility then the following must be specified in the manuscript: fertility status of female partner; and primary, secondary or other level of investigation of the man.
☑ If used in the manuscript, the term 'male factor' must be completely defined.

General aspects

☑ Patients were instructed to maintain 2–7 days of sexual abstinence before collecting a sample for investigation.
☑ Patients were informed about the importance of reporting any missed early ejaculate fractions, and men's answers were noted on the laboratory form.
NA For specimens not collected at the laboratory, patients were instructed to avoid cooling or heating of the semen sample during transport to the laboratory.
☑ Samples were kept at 37°C before initiation of and during the analysis in case of sperm motility assessment.
NA For samples collected adjacent to the laboratory, analysis was initiated after completion of liquefaction and within 30 min after ejaculation. If this was not done—and more importantly when some of the samples are collected in the laboratory and others are collected at home—it should be checked that this did not influence the data (and, if yes, that this effect must be included as a confounding factor in the statistical analysis).
☑ Liquefaction was first checked within 30 min after ejaculation.
☑ Volume was determined either by weighing or using a wide-bore volumetric pipette.
☑ Viscosity was measured using either a wide-bore pipette or a glass rod.
☑ All staff members who performed the analyses have been trained in basic semen analysis (ESHRE Basic Semen Analysis Course—or equivalent—and further in-house training) and participate regularly in internal quality control.
NA If more than one method can be recommended for a particular characteristic (e.g. to measure volume), only one should be used in a given study.

Sperm concentration assessment

☑ Semen aliquot to be diluted for sperm concentration assessment was taken with a positive displacement pipette (i.e. a 'PCR pipette') using a recommended diluent.
☑ Only standard dilutions were used (1:50, 1:20, or 1:10).
☑ Sperm concentration was assessed using haemocytometers with improved Neubauer ruling.
☑ Haemocytometers were allowed to rest for 10–15 min in a humid chamber to allow sedimentation of the suspended spermatozoa onto the counting grid before counting.
☑ Sperm counting was done using phase contrast microscope optics (200–400×).
☑ Comparisons were made between duplicate counts, and counts re-done when the

difference exceeded the acceptance limits.
☑ Typically at least 200 spermatozoa were counted in each of the duplicate assessments.

Sperm motility assessment
☑ Motility assessments were performed at 37℃+-0.5℃
☑ Motility assessments were done using phase contrast microscope optics (200–400×).
☑ Sperm motility was classified using a four-category scheme: rapid progressive, slow progressive, non-progressive, and immotile (World Health Organization, 1999; Bjo¨ rndahl et al., 2010; Barratt et al., 2011).
☑ Motility assessments were done in duplicate and compared; counts were re-done on new preparations when the difference between duplicates exceeded the acceptance limits.
☑At least 200 spermatozoa were assessed in each duplicate motility count.
☑ At least 5 microscope fields of view were examined in each duplicate count.

Sperm vitality assessment
☑ A validated supravital staining, appropriate to the type of microscope optics utilized, was used to assess sperm vitality.
☑ At least 200 spermatozoa were evaluated in each sample.
☑ Assessments were done under high magnification (×1000–1250) using a 100× high resolution oil immersion objective and bright field microscope optics (Ko¨ hler illumination).

Sperm morphology assessment
☑ Tygerberg Strict Criteria were used for the evaluation of human sperm morphology. Note: Another classification could be used for scientific studies with specific aims if the classification is described or referenced. Depending on the aim of the study, the evaluation of particular abnormal forms might be useful.
☑ Abnormalities are recorded for all four regions of the spermatozoon (head, neck/midpiece, tail and cytoplasmic residue) and the Teratozoospermia Index or 'TZI'was calculated (Bjo¨ rndahl et al., 2010; Barratt et al., 2011).
☑ If the laboratory claims to use Tygerberg Strict Criteria for the evaluation of human sperm morphology, then the laboratory must participate in an external quality assurance scheme to verify that its assessments comply with these criteria.
☑ The Papanicolaou staining method adapted for the assessment of human sperm morphology was used. For specific aims other staining methods could be used, but must then be declared and explained.
☑ At least 200 spermatozoa were assessed in each ejaculate.
☑ Assessments were done under high magnification (×1000–1250) using a 100× high resolution oil immersion objective and bright field microscope optics (Ko¨ hler illumination).

Other findings

☑ The presence of abnormal clumping (aggregates and agglutinates) was recorded.
☑ Abnormal viscosity was recorded.
☑The presence of inflammatory cells was recorded and reported if more than 1 million/ml.
☑ For the purpose of classifying infertility status (World Health Organization, 2010), antisperm antibodies were examined with a validated screening test

Analysing data
☑ The actual duration of sexual abstinence (in 'hours' or 'days') was recorded for each sample and included in the data reported in the manuscript.
☑ As a minimum in clinical studies, semen volume, sperm concentration, total number of spermatozoa/ejaculate, and abstinence time are given to reflect sperm production and output; only samples identified as having been collected completely can be included in the study.
☑ Confounding factors have been considered for statistical analysis: e.g. abstinence time and age, to evidence secular or geographical variations in sperm concentration or sperm count.
NA If appropriate, optional biochemical markers for prostatic, seminal vesicular and epididymal secretions were analysed and reported both as concentration and total amount.
☑ Signs of active infection/inflammation were noted and considered in the analysis of data in the study (e.g. inflammatory cells, impaired sperm motility, possibly also antisperm antibodies and reduction of secretory contributions).

Suppl. B

The participants were required to undergo 3–7 days of abstinence before providing samples of semen. Semen samples were collected by masturbation into a plastic tube in a dedicated room. Condoms and lubricants were prohibited. Samples were allowed to liquefy for less than 60 min prior to analysis. Sperm motility was classified into four grades (A, B, C, D) according to guidelines provided by the World Health Organization (WHO). The volume and pH value of ejaculate were measured, and other parameters such as sperm concentration, total sperm count, total motility, and the percentage of each motile sperm grade were determined with WLJY9000. Flow cytometry was used to evaluate sperm DNA fragmentation. Papanicolaou method was used for staining the sperm smear and sperm morphology was assessed using an optical microscope.

According to the fifth edition of the WHO laboratory manual for the examination and processing of human semen, asthenozoospermia was defined as a progressive motility of $< 32\%$ including the rapid and slow progressive motility, or a total motility of $< 40\%$ including progressive motility and non-progressive motility within 60 min of ejaculation over the previous three months. The total number or concentration of sperm and the percentage of morphologically normal spermatozoa was $\geq$ the lower reference limits.

Suppl. C

Consumption of different types of meat

Different types of meat	Normal	Asthenozoospermia
Red meat (g/day)	30.66±23.49	30.40±23.80
Poultry meat (g/day)	69.67±36.66	62.83±34.61
Processed meatballs/ sausage (g/day)	3.79±4.48	4.49±5.46
Ham/ bacon/ bologna (g/day)	2.67±5.07	2.78±5.51

Data were presented in mean ± standard deviation.

Association between total fish intake and asthenozoospermia.

	**Consumption of total fish (range, g/d)**			***P*-trend***
	**≤14.28**	**14.28-25.78**	**>25.78**	
Case/control	162/192	158/164	232/229	
Model 1 [a]	1.00 (reference)	1.11 (0.82, 1.51)†	1.11 (0.84, 1.47)	0.493
Model 2 [b]	1.00 (reference)	1.12 (0.82, 1.53)	1.06 (0.78, 1.43)	0.741
Model 3 [c]	1.00 (reference)	1.12 (0.82, 1.54)	1.13 (0.81, 1.58)	0.462

†Odds ratio (95% confidence interval) (all such value).

* Analysis of multiple logistic regression.

[a] Adjusted for age and BMI.

[b] Adjusted for age, BMI, smoking status, drinking status, total energy intake, household income, abstinence time, educational level, and physical activity.

[c] Further adjusted for cooking methods, total meat intake, total vegetable intake (based on model 2) (mutually adjusted for one another).

Suppl. D

```
proc ttest data;
class disease;
var variable;
run;

proc univariate data; var variable;
output out=data pctlpts=16.65 33.3 50 66.6 83.25
pctlpre=name pctlname=m1 d1 m2 d2 m3 noprint;
run;

proc means data mean std median qrange;
class disease;
var &vars;
run;

proc logistic data;
class disease classified variable;
model disease= independent variable confounding factors /aggregate scale=n plrl alpha=0.05;
run;
```
