# Supplementary material for: Associations between Meat and Vegetable Intake, Cooking Methods, and Asthenozoospermia: A Hospital-Based Case–Control Study in China
Source: Nutrients. 2022 May 7;14(9):1956. doi: 10.3390/nu14091956 (PMC9104795; doi:10.3390/nu14091956)
Supplement: Supplementary file 1 [file nutrients-14-01956-s001.zip › nutrients-1661098-supplementary/Supplementary/Supplementary tables_revised.pdf]

Supplementary table 1 Subgroup analysis of association between food intake and asthenozoospermia by age, BMI, and smoking status

		Controls	Cases	Level of food intake			*P*-trend*	*P*-interaction*
				Level 1	Level 2	Level 3		
Total meat	**Age**							
	<32 years	278	223	1.00 (reference)	**0.35 (0.19, 0.63)**	**0.36 (0.18, 0.70)**	**0.002**	0.249
	≥32 years			1.00 (reference)	0.89 (0.54, 1.46)	0.75 (0.42, 1.34)	0.333	
	**BMI**							
	<25 kg/m^2	241	201	1.00 (reference)	0.55 (0.30, 1.01)	0.65 (0.32, 1.33)	0.203	0.165
	≥25 kg/m^2	344	351	1.00 (reference)	0.70 (0.43, 1.13)	**0.50 (0.29, 0.88)**	**0.018**	
	**Smoking status**							
	Smokers	275	287	1.00 (reference)	**0.41 (0.23, 0.70)**	**0.32 (0.17, 0.59)**	**<0.001**	0.271
	Never smokers	310	265	1.00 (reference)	0.98 (0.58, 1.65)	1.11 (0.58, 2.12)	0.781	
Unprocessed meat	**Age**							
	<32 years	278	223	1.00 (reference)	**0.46 (0.24, 0.87)** †	**0.32 (0.17, 0.62)**	**<0.001**	0.122
	≥32 years	307	329	1.00 (reference)	1.28 (0.75, 2.21)	0.94 (0.54, 1.66)	0.562	
	**BMI**							
	<25 kg/m^2	241	201	1.00 (reference)	0.71 (0.37, 1.37)	0.74 (0.36, 1.49)	0.377	0.074
	≥25 kg/m^2	344	351	1.00 (reference)	1.07 (0.63, 1.82)	**0.57 (0.34, 0.96)**	**0.033**	
	**Smoking status**							
	Smokers	275	287	1.00 (reference)	0.59 (0.33, 1.06)	**0.41 (0.22, 0.73)**	**0.003**	0.234
	Never smokers	310	265	1.00 (reference)	1.15 (0.65, 2.05)	1.03 (0.56, 1.91)	0.902	
Processed meat	**Age**							
	<32 years	278	223	1.00 (reference)	1.64 (0.86, 3.19)	**1.88 (1.00, 3.61)**	0.099	0.395
	≥32 years	307	329	1.00 (reference)	1.33 (0.86, 2.06)	1.28 (0.81, 2.02)	0.404	
	**BMI**							
	<25 kg/m^2	241	201	1.00 (reference)	1.69 (0.98, 2.95)	1.58 (0.89, 2.82)	0.242	0.330
	≥25 kg/m^2	344	351	1.00 (reference)	1.24 (0.77, 2.01)	1.34 (0.83, 2.17)	0.282	
	**Smoking status**							
	Smokers	275	287	1.00 (reference)	1.10 (0.67, 1.84)	1.21 (0.73, 2.01)	0.453	0.690
	Never smokers	310	265	1.00 (reference)	**1.86 (1.10, 3.19)**	**1.79 (1.06, 3.06)**	0.121	
Vegetables	**Age**							
	<32 years	278	223	1.00 (reference)	0.85 (0.54, 1.36)	0.83 (0.48, 1.41)	0.530	0.729
	≥32 years	307	329	1.00 (reference)	0.97 (0.64, 1.46)	0.82 (0.51, 1.33)	0.396	

**BMI**							
<25 kg/m^2	241	201	1.00 (reference)	1.33 (0.80, 2.21)	1.75 (0.95, 3.24)	0.084	0.906
≥25 kg/m^2	344	351	1.00 (reference)	0.81 (0.54, 1.19)	**0.59 (0.38, 0.93)**	**0.023**	
**Smoking status**							
Smokers	275	287	1.00 (reference)	0.76 (0.48, 1.20)	0.72 (0.42, 1.20)	0.273	0.061
Never smokers	310	265	1.00 (reference)	1.18 (0.78, 1.80)	0.95 (0.58, 1.57)	0.764	

[*] Analysis of multiple logistic regression, adjusted for age, BMI, smoking status, drinking status, household income, abstinence time, educational level, physical activity, total energy intake, different cooking methods, total meat intake, unprocessed meat intake, processed meat intake and vegetables intake (mutually adjusted for one another).

† Odds ratio (95% confidence interval) (all such value).

Supplementary table 2 Subgroup analysis of association between different cooking methods and asthenozoospermia by age, BMI, and smoking status

			Frequency of different cooking methods for meat			*P*-trend*	*P*-int*
			~2-3 times/month	2~3 times/week	4 times/week~		
Deep-frying for meat							
**Age**							
<32 years	278	223	1.00 (reference)	0.46 (0.11, 1.90)	0.57 (0.14, 2.29)	0.694	0.093
≥32 years	307	329	1.00 (reference)	1.68 (0.55, 5.53)	1.75 (0.59, 5.67)	0.342	
**BMI**							
<25 kg/m^2	241	201	1.00 (reference)	0.56 (0.11, 2.81)	0.71 (0.15, 3.48)	0.971	**0.019**
≥25 kg/m^2	344	351	1.00 (reference)	1.26 (0.44, 3.81)	1.37 (0.49, 4.10)	0.509	
**Smoking status**							
Smokers	275	287	1.00 (reference)	1.67 (0.37, 8.29)	1.98 (0.45, 9.60)	0.278	0.654
Never smokers	310	265	1.00 (reference)	1.03 (0.35, 3.13)	1.08 (0.38, 3.26)	0.850	
Stewing for meat							
**Age**							
<32 years	278	223	1.00 (reference)	0.86 (0.49, 1.51)	0.85 (0.41, 1.76)	0.590	0.947
≥32 years	307	329	1.00 (reference)	1.02 (0.63, 1.65)	0.98 (0.53, 1.79)	0.984	
**BMI**							
<25 kg/m^2	241	201	1.00 (reference)	1.04 (0.57, 1.90)	0.95 (0.45, 2.01)	0.423	0.660
≥25 kg/m^2	344	351	1.00 (reference)	0.90 (0.57, 1.42)	0.94 (0.52, 1.71)	0.706	
**Smoking status**							
Smokers	275	287	1.00 (reference)	0.66 (0.38, 1.15)	0.86 (0.43, 1.71)	0.293	0.478
Never smokers	310	265	1.00 (reference)	1.31 (0.81, 2.14)	0.92 (0.49, 1.74)	0.549	
Broiling for meat							
**Age**							
<32 years	278	223	1.00 (reference)	0.75 (0.08, 6.82)	0.83 (0.09, 7.50)	0.913	0.688
≥32 years	307	329	1.00 (reference)	1.11 (0.19, 6.55)	0.83 (0.14, 4.82)	0.347	
**BMI**							
<25 kg/m^2	241	201	1.00 (reference)	2.33 (0.31, 47.93)	2.10 (0.28, 42.67)	0.925	0.911
≥25 kg/m^2	344	351	1.00 (reference)	0.23 (0.01, 1.72)	0.20 (0.01, 1.48)	0.105	
**Smoking status**							
Smokers	275	287	1.00 (reference)	-	-	0.439	0.567
Never smokers	310	265	1.00 (reference)	0.82 (0.19, 3.37)	0.65 (0.15, 2.65)	0.313	
Stir-frying for meat							
**Age**							
<32 years	278	223	1.00 (reference)	0.71 (0.43, 1.16)	0.84 (0.39, 1.80)	0.220	0.172
≥32 years	307	329	1.00 (reference)	0.99 (0.64, 1.53)	1.35 (0.68, 2.70)	0.751	
**BMI**							
<25 kg/m^2	241	201	1.00 (reference)	0.95 (0.56, 1.61)	1.07 (0.45, 2.56)	0.895	0.278
≥25 kg/m^2	344	351	1.00 (reference)	0.77 (0.51, 1.16)	1.07 (0.57, 2.01)	0.347	
**Smoking status**							
Smokers	275	287	1.00 (reference)	0.84 (0.53, 1.34)	1.24 (0.62, 2.48)	0.698	0.492
Never smokers	310	265	1.00 (reference)	0.83 (0.53, 1.30)	0.84 (0.39, 1.82)	0.562	

	Frequency of different cooking methods for vegetables			*P*-trend*	*P*-int*
	~2-3 times/month	2~3 times/week	4 times/week~		
Deep-frying for vegetables					
**Age**					
<32 years	1.00 (reference)	2.18 (0.37, 15.02)	2.88 (0.54, 18.31)	0.179	0.809
≥32 years	1.00 (reference)	0.61 (0.14, 2.41)	0.79 (0.20, 2.75)	0.892	
**BMI**					
<25 kg/m^2	1.00 (reference)	2.18 (0.38, 13.77)	1.74 (0.35, 9.77)	0.630	0.796
≥25 kg/m^2	1.00 (reference)	0.67 (0.16, 2.67)	1.17 (0.31, 4.21)	0.461	
**Smoking status**					
Smokers	1.00 (reference)	0.77 (0.13, 4.40)	1.60 (0.29, 8.02)	0.237	0.861
Never smokers	1.00 (reference)	1.11 (0.28, 4.36)	0.95 (0.27, 3.42)	0.860	
Stewing for vegetables					
**Age**					
<32 years	1.00 (reference)	0.86 (0.55, 1.34)	0.81 (0.49, 1.35)	0.398	0.312
≥32 years	1.00 (reference)	0.72 (0.49, 1.05)	0.82 (0.51, 1.33)	0.140	
**BMI**					
<25 kg/m^2	1.00 (reference)	**0.62 (0.39, 0.98)**	0.73 (0.42, 1.25)	0.070	0.092
≥25 kg/m^2	1.00 (reference)	0.87 (0.60, 1.26)	0.88 (0.56, 1.37)	0.457	
**Smoking status**					
Smokers	1.00 (reference)	0.75 (0.50, 1.13)	0.80 (0.47, 1.33)	0.190	0.089
Never smokers	1.00 (reference)	0.78 (0.52, 1.17)	0.81 (0.50, 1.29)	0.237	
Broiling for vegetables					
**Age**					
<32 years	1.00 (reference)	**4.75 (1.04, 35.37)**	**5.25 (1.21, 38.32)**	**0.049**	0.541
≥32 years	1.00 (reference)	0.43 (0.08, 2.05)	0.64 (0.12, 2.90)	0.987	
**BMI**					
<25 kg/m^2	1.00 (reference)	2.55 (0.52, 19.02)	3.38 (0.74, 24.01)	0.113	0.625
≥25 kg/m^2	1.00 (reference)	1.17 (0.30, 4.85)	1.49 (0.40, 5.94)	0.377	
**Smoking status**					
Smokers	1.00 (reference)	0.72 (0.07, 7.29)	1.45 (0.15, 13.93)	0.147	0.693
Never smokers	1.00 (reference)	2.09 (0.66, 7.48)	1.91 (0.63, 6.68)	0.333	
Stir-frying for vegetables					
**Age**					
<32 years	1.00 (reference)	0.96 (0.62, 1.47)	0.62 (0.30, 1.24)	0.398	0.092
≥32 years	1.00 (reference)	1.00 (0.68, 1.46)	1.42 (0.63, 3.29)	0.737	
**BMI**					
<25 kg/m^2	1.00 (reference)	0.92 (0.58, 1.46)	0.56 (0.21, 1.38)	0.402	0.084
≥25 kg/m^2	1.00 (reference)	0.98 (0.68, 1.41)	1.18 (0.62, 2.28)	0.874	
**Smoking status**					
Smokers	1.00 (reference)	0.99 (0.66, 1.51)	0.90 (0.37, 2.13)	0.894	0.770
Never smokers	1.00 (reference)	0.95 (0.64, 1.41)	0.88 (0.45, 1.70)	0.713	

Supplementary table 2 *continued*

			Frequency of different cooking methods for meat			*P*-trend	*P*-int
			~2-3 times/month	2~3 times/week	4 times/week~		
Steaming for meat							
**Age**							
<32 years	278	223	1.00 (reference)	0.84 (0.48, 1.46)	-	0.545	0.125
≥32 years	307	329	1.00 (reference)	1.01 (0.22, 4.50)	0.78 (0.18, 3.32)	0.482	
**BMI**							
<25 kg/m^2	241	201	1.00 (reference)	-	-	0.552	0.172
≥25 kg/m^2	344	351	1.00 (reference)	0.54 (0.10, 2.54)	0.52 (0.10, 2.35)	0.411	
**Smoking status**							
Smokers	275	287	1.00 (reference)	0.56 (0.06, 5.06)	0.81 (0.09, 7.07)	0.604	**0.045**
Never smokers	310	265	1.00 (reference)	0.84 (0.10, 5.86)	0.58 (0.07, 3.94)	0.235	

	Frequency of different cooking methods for vegetables			*P*-trend	*P*-int
	~2-3 times/month	2~3 times/week	4 times/week~		
Raw vegetables					
**Age**					
<32 years	1.00 (reference)	0.66 (0.36, 1.20)	0.73 (0.40, 1.31)	0.259	0.206
≥32 years	1.00 (reference)	0.82 (0.49, 1.38)	0.62 (0.37, 1.03)	0.084	
**BMI**					
<25 kg/m^2	1.00 (reference)	0.78 (0.43, 1.42)	0.68 (0.37, 1.24)	0.217	0.270
≥25 kg/m^2	1.00 (reference)	0.73 (0.43, 1.24)	0.63 (0.37, 1.05)	0.082	
**Smoking status**					
Smokers	1.00 (reference)	0.74 (0.41, 1.32)	0.68 (0.38, 1.20)	0.184	0.663
Never smokers	1.00 (reference)	0.70 (0.41, 1.21)	0.62 (0.36, 1.06)	0.081	

BMI, body mass index; int, interaction.

* Analysis of multiple logistic regression, adjusted for age, BMI, smoking status, drinking status, household income, abstinence time, educational level, physical activity, total energy intake, different cooking methods, total meat intake and vegetables intake.

† Odds ratio (95% confidence interval) (all such value).

Supplementary table 3 Odds ratio (95% CI) for risk of asthenozoospermia according to combined effect of meat intake and cooking methods

Total meat intake (g/day)	Deep-frying for meat		Stewing for meat		Broiling for meat		Stir-frying for meat		Steaming for meat	
	<2-3 times a month	⩾2-3 times a month	<2-3 times a month	⩾2-3 times a month	<2-3 times a month	⩾2-3 times a month	<2-3 times a month	⩾2-3 times a month	<2-3 times a month	⩾2-3 times a month
N (%)	364	773	50	1087	139	998	16	1121	644	493
T1 (≤89.43)	1.00 (ref)	1.00 (0.67, 1.50)	1.00 (ref)	1.40 (0.73, 2.74)	1.00 (ref)	0.92 (0.54, 1.56)	1.00 (ref)	1.19 (0.39, 3.66)	1.00 (ref)	1.11 (0.73, 1.69)
T2 (89.43-116.44)	0.78 (0.45, 1.37)	**0.57 (0.35, 0.90)**	-	0.86 (0.43, 1.76)	0.58 (0.25, 1.32)	0.59 (0.33, 1.05)	0.92 (0.03, 27.83)	0.81 (0.26, 2.52)	**0.59 (0.38, 0.91)**	0.75 (0.47, 1.19)
T3 (>116.44)	0.65 (0.34, 1.23)	**0.53 (0.32, 0.89)**	1.73 (0.27, 15.23)	1.74 (0.36, 1.54)	0.32 (0.11, 0.87)	0.59 (0.29, 1.03)	-	0.74 (0.23, 2.34)	**0.55 (0.33, 0.91)**	0.64 (0.38, 1.07)

T, tertile; ref, reference; "-" represented no valid effect value due to the narrow distribution.

Analysis of multiple logistic regression, adjusted for age, BMI, smoking status, drinking status, household income, abstinence time, educational level, physical activity, total energy intake, different cooking methods, and vegetables intake.

Table 4 Association between different cooking methods for vegetables and asthenozoospermia

	Frequency of different cooking methods for vegetables			P-trend*
	~2-3 times/month	2~3 times/week	4 times/week~	
**Deep-frying for vegetables**				
Case/control	508/531	34/45	10/9	
Model 1 [a]	1.00 (reference)	0.66 (0.24, 1.82)	0.83 (0.32, 2.08)	0.923
Model 2 [b]	1.00 (reference)	0.82 (0.29, 2.30)	1.07 (0.40, 2.79)	0.669
Model 3 [c]	1.00 (reference)	0.98 (0.34, 2.84)	1.28 (0.48, 3.44)	0.441
**Stewing for vegetables**				
Case/control	119/137	254/289	179/159	
Model 1 [a]	1.00 (reference)	0.78 (0.59, 1.03)	0.79 (0.57, 1.09)	0.065
Model 2 [b]	1.00 (reference)	0.79 (0.60, 1.05)	0.85 (0.60, 1.19)	0.140
Model 3 [c]	1.00 (reference)	0.76 (0.58, 1.01)	0.82 (0.58, 1.15)	0.086
**Broiling for vegetables**				
Case/control	486/496	58/76	8/13	
Model 1 [a]	1.00 (reference)	1.51 (0.45, 3.09)	1.44 (0.60, 3.67)	0.255
Model 2 [b]	1.00 (reference)	1.43 (0.54, 4.10)	1.85 (0.73, 5.13)	0.116
Model 3 [c]	1.00 (reference)	1.55 (0.57, 4.59)	1.97 (0.75, 5.67)	0.107
**Stir-frying for vegetables**				
Case/control	30/38	131/148	391/399	
Model 1 [a]	1.00 (reference)	0.92 (0.70, 1.21)	0.84 (0.51, 1.39)	0.416
Model 2 [b]	1.00 (reference)	0.98 (0.74, 1.30)	0.93 (0.55, 1.54)	0.790
Model 3 [c]	1.00 (reference)	0.97 (0.73, 1.28)	0.91 (0.54, 1.53)	0.724
**Raw vegetables**				
Case/control	265/315	198/201	89/69	
Model 1 [a]	1.00 (reference)	0.76 (0.52, 1.10)	**0.66 (0.46, 0.95)**	**0.025**
Model 2 [b]	1.00 (reference)	0.78 (0.53, 1.14)	0.69 (0.48, 1.00)	0.054
Model 3 [c]	1.00 (reference)	0.75 (0.51, 1.11)	**0.67 (0.45, 0.98)**	**0.041**

[*] Analysis of multiple logistic regression.

† Odds ratio (95% confidence interval) (all such value).

[a] Adjusted for age and BMI.

[b] Adjusted for age, BMI, smoking status, drinking status, total energy intake, household income, abstinence time, educational level, and physical activity.

[c] Further adjusted for total meat intake, different cooking methods for meat, and vegetables intake (based on model 2).

Supplementary table 5 Odds ratio (95% CI) for risk of asthenozoospermia according to combined effect of vegetables intake and cooking methods

Vegetable intake (g/day)	Deep-frying for vegetables		Stewing for vegetables		Broiling for vegetables		Stir-frying for vegetables		Raw vegetables	
	<2-3 times a month	≥2-3 times a month	<2-3 times a month	≥2-3 times a month	<2-3 times a month	≥2-3 times a month	<2-3 times a month	≥2-3 times a month	<2-3 times a month	≥2-3 times a month
N (%)	854	292	77	1060	627	510	20	1117	260	877
T1 (≤121.25)	1.00 (ref)	1.23 (0.69, 2.19)	1.00 (ref)	1.48 (0.68, 3.38)	1.00 (ref)	0.88 (0.57, 1.36)	1.00 (ref)	0.71 (0.19, 2.73)	1.00 (ref)	0.98 (0.62, 1.55)
T2 (121.25-207.07)	0.93 (0.66, 1.31)	1.08 (0.67, 1.74)	1.69 (0.56, 5.22)	1.32 (0.60, 3.03)	0.87 (0.59, 1.29)	0.93 (0.60, 1.42)	0.86 (0.10, 7.16)	0.67 (0.17, 2.57)	0.80 (0.44, 1.46)	1.00 (0.63, 1.57)
T3 (>207.07)	0.89 (0.61, 1.32)	0.80 (0.50, 1.30)	1.83 (0.56, 6.31)	1.16 (0.52, 2.71)	0.83 (0.54, 1.28)	0.76 (0.48, 1.20)	1.82 (0.15, 46.37)	0.59 (0.15. 2.28)	0.86 (0.46, 1.62)	0.88 (0.54, 1.44)

T, tertile; ref, reference; “-” represented no valid effect value due to the narrow distribution.

Analysis of multiple logistic regression, adjusted for age, BMI, smoking status, drinking status, household income, abstinence time, educational level, physical activity, total energy intake, different cooking methods, and total meat intake.
