# Supplementary figures and images for: Associations between Meat and Vegetable Intake, Cooking Methods, and Asthenozoospermia: A Hospital-Based Case–Control Study in China
Source: Nutrients. 2022 May 7;14(9):1956. doi: 10.3390/nu14091956 (PMC9104795; doi:10.3390/nu14091956)

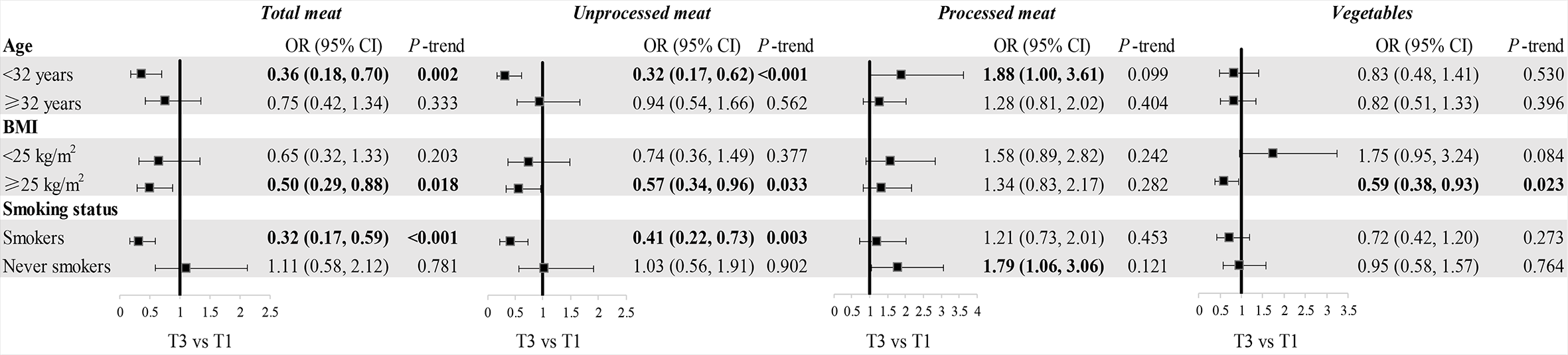

Supplement: Supplementary file 1 [file nutrients-14-01956-s001.zip › nutrients-1661098-supplementary/Supplementary/Supplementary Figure 1.tif]
